# Left ventricular volume regulation in heart failure with preserved ejection fraction

**DOI:** 10.1002/phy2.7

**Published:** 2013-06-26

**Authors:** Peter L M Kerkhof, J Yasha Kresh, John K-J Li, Guy R Heyndrickx

**Affiliations:** 1 Department Physics & Medical Technology, VU University Medical CenterAmsterdam, The Netherlands; 2 Cardiothoracic Surgery, Medicine and Biomedical Engineering, Drexel UniversityPhiladelphia, PA; 3 Department Biomedical Engineering, Rutgers University and UMDNJ-Robert Wood Johnson Medical SchoolPiscataway, NJ; 4 Cardiovascular Center Aalst, OLV ClinicAalst, Belgium

**Keywords:** Ejection fraction, heart failure, left ventricular function, pathophysiology

## Abstract

Ejection Fraction (EF) has attained the recognition as indicator of global ventricular performance. Remarkably, precise historical origins promoting the apparent importance of EF are scant. During early utilization EF has been declared a gold standard for the evaluation of the heart as a pump. In contrast, during the last two decades, clinicians have developed a measure of doubt in the universal applicability of EF. This reluctance lead to the introduction of a new and prevalent syndrome in which heart failure (HF) is diagnosed as having a preserved EF (pEF). We examine the existing criticism regarding EF, and describe a novel avenue to characterize ventricular function within the unifying framework of cardiac input–output volume regulation. This approach relates end-systolic volume (ESV) to end-diastolic volume (EDV), and derives for a subgroup matching pEF criteria a distinct pattern in the ESV–EDV domain. In patients with pEF (*n* = 34), a clear difference (*P* < 0.0004) in the slope of the regression line for ESV versus EDV was demonstrated compared to control patients with EF < 50% (*n* = 29). These findings are confirmed by analysis of data presented in two independent publications. The volume regulation approach proposed employs primary end-point determinants (such as ESV and EDV) rather than derived quantities (e.g., the ratio EF or its differential parameter, that is, stroke volume) and confirms a distinct advantage over the classical Starling curve. Application of the ESV-EDV-construct provides the basis and clarifies why some patients present as HFpEF, while others have reduced EF.

## Introduction

### Historical background of contractile performance indexes related to EF

Various imaging techniques allow for the measurement of end-systolic volume (ESV) and end-diastolic volume (EDV), both in left ventricle (LV) and right ventricle (RV). Stroke volume (SV) is the difference between EDV and ESV, assuming absence of valvular regurgitation. Ejection Fraction (EF) is defined as SV divided by EDV, resulting in a plain dimensionless number. Consequently, determination of EF is rather simple because calibrated volumetric measurements are not required and body mass does not matter. To allow comparisons of persons of different size, the calibrated values for ESV and EDV often require further correction for body surface area (BSA, expressed as m^2^). Adjustment of volume measurements for BSA is denoted as index (I) using the affix I to ESV and EDV.

In 1934 a new cardiac performance indicator was introduced by taking the ratio of (total) heart volume and SV (Lysholm et al. 1934). Motivated mostly by intuition, the authors proposed a new cardiac metric that was a preamble to EF which was not in use at the time. In retrospect, their approach clearly points toward EF, apart from the fact that they considered total heart volume rather than just LV or RV volume. The dye dilution technique was introduced in 1951 to enable the metric of EDV divided by SV (Bing et al. 1951). Note that both studies document the inverse of the EF metric. Later, a relationship between EF and myocardial contractility was established, showing that a low value for EF corresponds to poor LV function (Bartle et al. 1965). Since that period, the index EF gradually became a gold standard for assessing functioning of LV and RV. The increasing applicability of various types of dilution techniques, followed by the introduction of angiography, echocardiography, and MRI may have further encouraged the pervasive utility of EF, still in use today.

As EF reflects a measure of the efficiency of forward pumping, researchers wondered how the fractional volume displacement (cf. volume strain) in healthy individuals can reach 70% or even higher, while LV diameter shortening remains relatively small. Mathematical considerations revealed that helical fiber orientation and mechanical syncytium explain large values of EF (i.e., >50%), despite a (one dimensional) fractional segment shortening limited to maximally 15 or 20% (Sallin 1969). EF became an easily computable, convenient, and descriptively compelling index because the numerical value ranges from almost zero to near 100 (when expressed as a percentage). Subsequently, EF has been compared with other evolving performance indicators such as circumferential shortening velocity, the maximum time-derivative of LV pressure (d*P*/d*t*)_max_, and more recently end-systolic elastance (Emax). Furthermore, allometric studies demonstrated that the normal value for all mammalian species is about the same, that is, around 65% for healthy subjects, suggesting that EF remains invariant of body mass (Li 1996).

Recently, some investigators invoked a more adaptive holistic approach aiming to unify metabolism, growth and morphogenesis, and sense physiology into a general dynamic theory of open systems (Kresh 2006). While appreciating the overwhelming complexity of cardiac function at the cellular level as well as the various pathways of (patho)physiologic adaptation, we confine ourselves in this analysis to the study of macroscopic parameters such as ESV and EDV, including the ratio EF.

**Figure 1:**
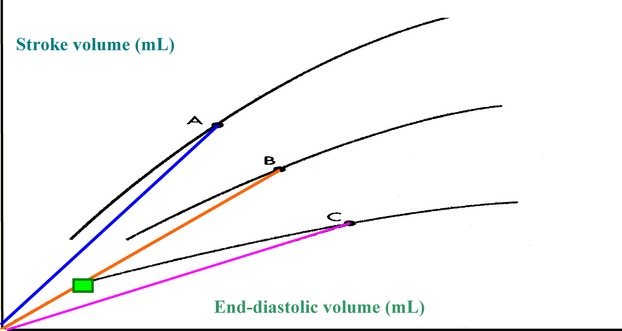
Schematic examples of Starling curves. Ejection Fraction (EF) can be assessed from the curve by taking the slope of the line connecting the origin with the actual operating point (A, B, or C in this example, referring to curves with high, normal, and low contractility, respectively). Note that EF always decreases when end-diastolic volume (EDV) rises.

It is important to recognize that within the framework of the *classical Starling curve* (which essentially relates SV to EDV), the parameter EF emerges in a natural manner by being the slope of the line connecting the operating point on the prevailing curve (e.g., points A, B, and C in Fig. 1) with the origin of the graph. Note that for each Starling curve the value of EF decreases as EDV rises, implying that preload recruitable SV results in a counterintuitive decreased performance as judged by the index EF. Moreover, one can easily envision an *impaired* SV–EDV function curve in which an operating point (C in Fig. 1) is transferred to a new value such that the computed EF assumes a relatively normal value (at low EDV near the square in Fig. 1) and compares favorably with the operating point (B in Fig. 1) on a curve corresponding with a *normal* heart. This striking observation further challenges the purported relevance regarding the universality of EF.

### Previous criticisms regarding EF

**Figure 2:**
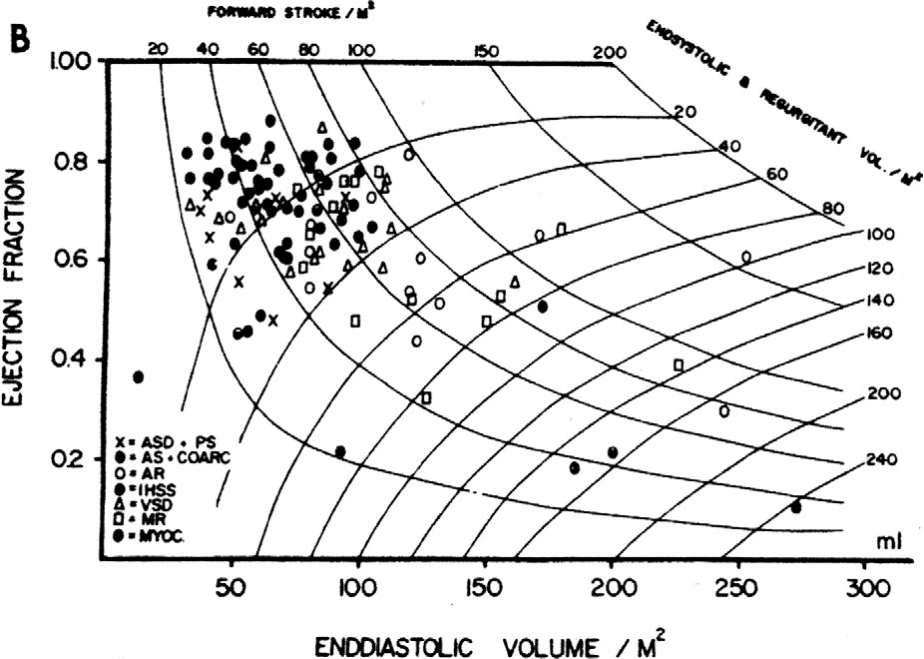
Ejection Fraction (EF) versus End-Diastolic Volume Index (EDVI) in 123 patients, reproduced from Hugenholtz and Wagner (1970) (their Figure 121B). All relationships are nonlinear. AS, aorta stenosis; ASD, atrial septum defect; AR, aortic regurgitation; COARC, coarctatio aortae; IHSS, idiopathic hypertrophic subaortic stenosis; MR, mitral regurgitation; MYOC, myocarditis; PS, pulmonic stenosis; VSD, ventricular septum defect. (This material is reproduced with permission of John Wiley & Sons, Inc.).

In 1978 researchers proposed that EF at SV = 70 mL is a better index to separate normals from cardiomyopathy patients (Bowyer and Asato 1978). As far as we could trace, this report was the first critical note emphasizing that inclusion of absolute measures of LV volume may be more informative than the ratio EF alone. In fact, over the years quite a number of clinicians have expressed their concerns regarding the pretended universal validity of EF as the preferred indicator of LV performance. Various investigators have attempted to relate EF to volumetric measures, for example, to end-diastolic volume index (EDVI) (Hugenholtz and Wagner 1970; Hamilton et al. 1972; Robotham et al. 1991) and they found nonlinear (e.g., hyperbolic) relationships. Figure 2 shows the graphical representation relating EF to EDVI for patients (*n* = 123) representing a wide spectrum of cardiac pathologies, where nonlinear iso-stroke volume index (SVI) and iso-end-systolic volume index (ESVI) curves are inscribed (Hugenholtz and Wagner 1970). The authors concluded: *It thus is obviously erroneous to consider the ejection fraction per se as a measure of the adequacy of ventricular function. Yet, under resting circumstances, and with knowledge of the end-diastolic pressure and volume as well as of the peripheral resistance, the ejection fraction is a very useful first approximation of the state of the myocardium* (Hugenholtz and Wagner 1970). In another study (dating back to 1991) it was concluded: *Thus, EF, although a relatively simple measure that is intuitively easily comprehended, is an extremely complex parameter describing the entire cardiovascular system and requires additional study* (Robotham et al. 1991). As we will demonstrate in the next section, the graphical plot of ESVI versus EDVI displays clear advantages because all interrelations are visible as linearities, including EF. This transformed functional representation will also illustrate the limited usefulness of EF.

### The unifying framework for cardiac volume regulation

**Figure 3:**
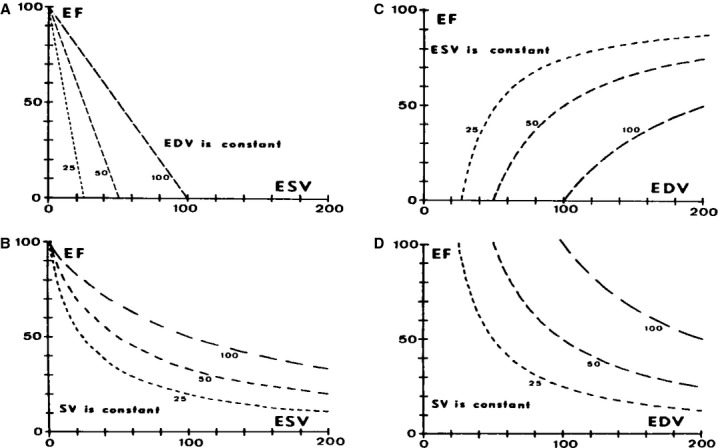
Theoretical relationships between Ejection Fraction (EF) and end-systolic volume (ESV) or end-diastolic volume (EDV), respectively. Traces refer to various constant levels for ESV, EDV, or SV.

In general, the use of a quotient (i.e., relative metrics) such as EF implies certain limitations. Clearly, the ratio of any pair of numbers can be the same for an infinite series of numbers, for example, 25/50 = 115/230, etc. Therefore, there is no a priori reason to believe that EF carries universally valid information on cardiac performance, let alone the embodiment of a gold standard. This crucial point can be best appreciated when considering the paradigm of volume regulation of the ventricle (Kerkhof 1984). Figure 1 shows the classical Starling-type of curve, typically relating SV to EDV. However, we proposed to plot ESV(I) versus EDV(I) and hence coined the term *Alternative Starling Curve* (ASC) (Kerkhof 1984, 2000; Kerkhof and Kresh 1990; Beringer and Kerkhof 1998). In earlier investigations we assessed both LV and RV volume (V) using in vivo labeled erythrocytes as tracer (15 mCi 99mTc) for gated equilibrium studies in 75 patients with angina pectoris, and found linear relationships for ESVI versus EDVI with *r* = 0.92 for LV and *r* = 0.95 for RV, respectively (Beringer and Kerkhof 1998). Elsewhere we have documented similar relationships which formed the basis for the description of volumetric regulation of the cardiodynamics of both LV and RV in humans (Kerkhof 1984, 2000; Kerkhof and Kresh 1990; Beringer and Kerkhof 1998) and for the LV in dogs (Kerkhof et al. 1998). Independent studies by others support the notion of ASC, for example, for better understanding of the interaction between LV and continuous cardiac assist, the effect of different working conditions and support levels on LV PV-loops was investigated in acute animal experiments; linear relationships were found for both individual and for pooled data collected in seven sheep, for example, ESVI = 0.845 EDVI − 15.21, *r*^2^ = 0.924, *P* < 0.0001, *n* = 200 (Moscato et al. 2007). In summary, the following linear equation generally applies:


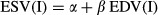
(1)

with Pearson's coefficient of correlation *r*, where α (mL or mL/m^2^) is the volume axis intercept and β (dimensionless) the slope (cf. gain function system parameter) of the regression line. Elsewhere, we have shown that the ASC yields a higher *r* than the one for the corresponding Starling curve if β > 0.5 (Beringer and Kerkhof 1998). Equation (1) can be employed to formulate the mathematical behavior of derived parameters, such as EF (here not expressed as percentage but as a fraction between 1.0 and zero) (Kerkhof 1984):


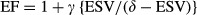
(2)

where yielding *δ* = *α* − EDV_ave_(1 − *r*^2^) *β*/*r*^2^ while EDV_ave_ is the average value of EDV for the population under consideration. It is important to point out that EF versus ESV is essentially nonlinear. The derived parameter EF, while by definition a function of both ESV and EDV (namely EF = 1 − ESV/EDV), can be conveniently expressed as uniquely related to ESV; by virtue of the rather simple ESV–EDV regression, this results in equation 2. To adhere to fundamentals, however, it is required to invoke all features of the particular ESV-EDV graph under investigation, namely the pertinent values of intercept (α), slope (β), correlation coefficient (*r*), and EDV_ave_. The newly introduced definitions of γ and δ render the expression more readable while encompassing all these contributing elements. Clearly, EF depends explicitly on the prevailing value of ESV in a nonlinear fashion, as can be gleaned from the interpretation of equation 2. If indeed α and/or β are different in HFpEF patients compared to a control group, then this distinction will translate into diverging patterns for the EF–ESV relationship. Such a difference would explain why EF acts as the key index in some patients whereas it cannot serve this purpose in others. The typical Starling curve relates SV (i.e., the *difference* between EDV and ESV) to EDV. Therefore, the relationship is amenable to spurious correlations as SV depends directly on EDV. Consequently, it is more correct to plot both basic components in a graph, yielding the ESV versus EDV representation. The latter graph easily translates into the familiar EF–ESV relationship (e.g., Figs. 3, 5, 6) on the basis of equation 2.

The relation between EF and ESV can be appreciated by analyzing simple graphical representations. Based on the definition formula for EF, it is self-evident that SV, ESV, EDV, and EF are interrelated. Figure 3A–D illustrate how EF depends on ESV and EDV when either SV or EDV or ESV is constant. When EDV remains constant, the relationship between EF and ESV is by definition a linear one. Remarkably, EF versus ESV is fairly linear (for EF >20%) even when EDV is allowed to vary (Kerkhof et al. 1981). Strictly speaking this finding implies that ESV embodies similar information as EF. However, it was also documented that this simple conclusion is not universally applicable (Kerkhof et al. 1981). Indeed, in the case of chronic beta-adrenergic blockade the relationship becomes diffuse, thus deviating from the one-to-one connection (Kerkhof et al. 1981). In fact, additional circumstances may apply where this apparently trivial connection does not hold true. Apart from this implied clinical ambiguity, several mathematically based observations remain proven, for example, reducing ESV for whatever reason will always lead to an increase of EF, regardless of whether EDV (Fig. 3A) or SV is constant (Fig. 3B). An increase of EDV will result in an augmented EF when ESV is constant (Fig. 3C) but not when SV is constant (Fig. 3D). As the (patho)physiological range of SV is limited, an increased EDV will ultimately imply a rise of ESV with concomittant reduction of EF. In accordance with equation 2, Figure 3B suggests that EF and ESV are inversely related in a more or less linear fashion.

Nearly two decades ago a new and prevalent (i.e., more than 2.5 million patients in the United States of America alone) syndrome was identified where heart failure (HF) is diagnosed with preserved EF (HFpEF) (Norman et al. 2011). This syndrome was previously referred to by the more mechanistic term *diastolic HF* (DHF), but nowadays some investigators doubt the primary cause as being strictly diastolic. Apart from the remarkable fact that a subset of an existing cardiac disorder was identified as a novel syndrome referring to millions of patients worldwide, the major impact from this counterintuitive conclusion is the notion that the clinical community is openly forced to accept that EF has lost its acclaimed universal applicability as index for characterizing ventricular performance. It is perplexing to learn that the traditional performance index EF does not apply to half of all patients with HF. While the practical value of EF in this subset of patients has been challenged, it is nonetheless surprising to witness that the same index EF is still being used for evaluation of a protocol (maximum dose 16 μg/kg.min i.v. dobutamine) in patients with known HFpEF when compared with controls, while the primary outcome was measured as a percent wise change in EF (Norman et al. 2011). Indeed, in patients where EF was demonstrated not to be a useful indicator of clinical status, the same index should not be welcomed to evaluate effects of interventions affecting performance.

The ASC approach readily explains the mathematical basis for the possible relevance of EF. This notion is particularly important when considering subgroups of patients that exhibit certain constraints in terms of volumetric characteristics. Based on the current ESC guidelines (Paulus et al. 2007), besides an LV end-diastolic pressure (EDP) >16 mmHg, the boundary conditions for the category referring to the HFpEF syndrome include EF >50% (i.e., in terms of the ASC: ESVI <0.5 EDVI) combined with EDVI <97 mL/m^2^. Thus, for pEF the two volumetric restrictions imply that the corresponding regression coefficients α and β in equation 1 have clear consequences for the predicted value (see eq. (2)) of EF compared to controls.

## Methods

### Patient data analysis

This study examines three sets of patient data allowing for the estimation of the ASC (and the resulting EF–ESV relationship) pertaining to various conditions. One study (A) addresses the issue of pEF compared to a control group, whereas the other two studies are derived from the literature and concern survival (range 15–165 months) in 605 adults under 60 years of age after myocardial infarction (MI) (B), and 1-year survival of 18 infants with cardiomyopathy (C), respectively.

Retrospective analysis of our 63 patients with left ventricular end-diastolic pressure (LVEDP) >16 mmHg was carried out. Data sets were angiographically collected by one of us (G. R. H.) at the Cardiovascular Center in Aalst, Belgium. Patients taking beta-blockers were excluded because beta-blockade has been demonstrated to exert a significant effect on EF-ESV (Kerkhof et al. 1981). Ventriculograms are recorded in right anterior oblique and left anterior oblique views on cine film at 30–60 frames/sec, and radiographic contrast agent is injected at rates of 7–15 mL/sec for a total volume of 30–50 mL. Cine frames are selected at end-diastole and end-systole. For the calculation of LV volume, the outermost margin of visible radiographic contrast is traced, and computed using long-axis (L) and short-axis measurements or area (A)-length measurements (V = 8A^2^/3πL) using an ellipsoid approximation for ventricular shape (Dodge et al. 1960). Corrections are made for magnification of the ventricular image onto the image intensifier, yielding ESV and EDV which are indexed for BSA. Intraventricular pressures were recorded through the pigtail catheter used for performing LV angiography. After debubbling, the catheter was connected through short stiff tubing, to a Statham P23Db strain gauge manometer (Gould Instruments, Oxnard, CA), previously calibrated against an external mercury manometer. Timing of the LVEDP corresponds to the *R*-wave intersection with the LV pressure tracing. Using reported P and V criteria for pEF we define a subgroup consisting of 34 patients (Paulus et al. 2007). Subsequently we compare the pEF group with the remaining 29 patients serving as controls with reduced EF (rEF, i.e., EF <50%).

### Statistical analysis

Pearson's coefficient of correlation for linear regression analysis regarding ESV versus EDV is denoted as *r*, while *r′* is employed for EF versus ESV. Significance of differences between regression lines is calculated using pooled covariances.

## Results

**Figure 4:**
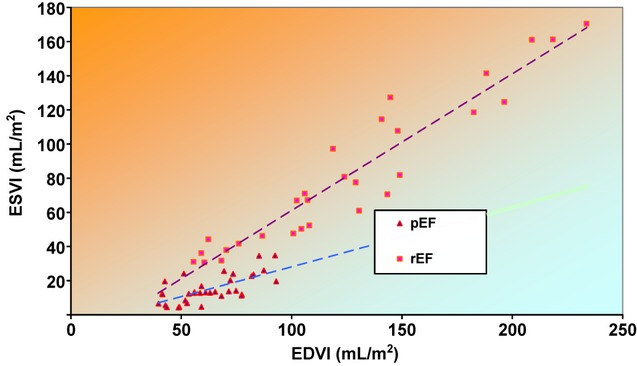
Graph of ESVI versus EDVI with linear regression lines for our patient groups (pEF triangles and control as squares).

**Figure 5:**
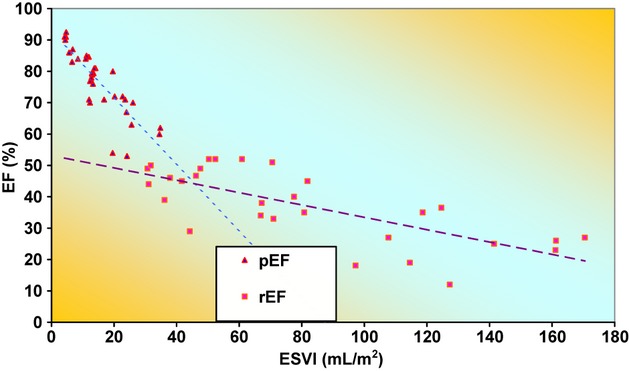
Graph of EF versus ESVI for our patient groups (pEF triangles and control as squares).

(A) Our findings regarding ESVI versus EDVI, and EF versus ESVI are presented in Figures 4 and 5, respectively. Corresponding linear regression coefficients are calculated, along with values for α, β, γ, and *δ* for both groups:


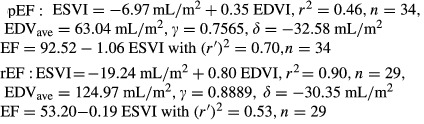


**Figure 6:**
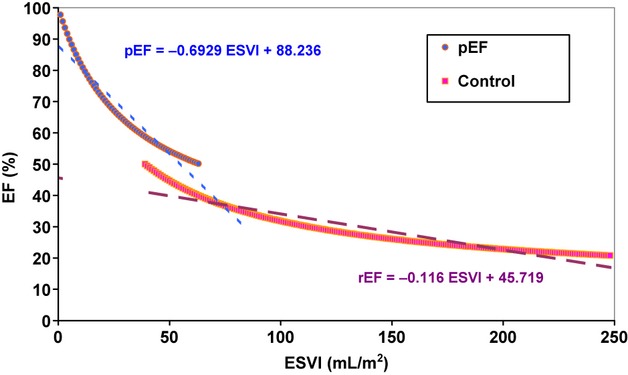
Predicted representation of EF versus ESVI for our patient groups based on the analytical expression given in equation 2 (along with linearized fitting curves).

**Figure 7:**
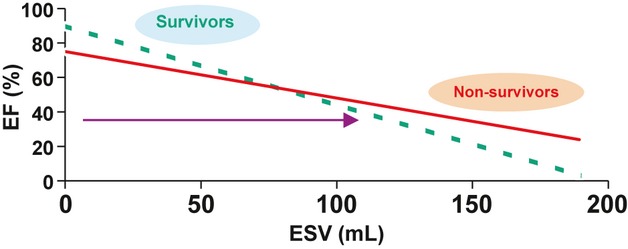
Schematic image, redrawn after a graphical representation of two groups of patients, all with myocardial infarction. The *r′* is −0.78 for both linear regression lines. The subgroup of nonsurvivors (*n* = 120) shows a slope for EF versus ESV which is less steep (*P* < 0.001) than for the survivors (*n* = 485). Arrow indicates EF level of 35%. Derived from White et al. (1987).

Based on the pooled analysis of variance, the slope for the pEF group in the ASC representation was significantly (*P* < 0.0004) lower than for the controls (Fig. 4). This resulted in two distinct EF–ESVI relationships with significantly (*P* < 0.0001) different slopes (see eq. (2), Figs. 5 and 6). In Figure 5 for simplicity we fitted linear regression, although equation 2 predicts a nonlinear relationship as shown in Figure 6. It must be noted that a polynomial fit offers no advantages over the precise curvilinear description given by the simple analytical expression formulated in equation 2. Most importantly, equation 2 incorporates not only the regression coefficients α and β but also the actual value of the correlation coefficient *r* of the ASC. In summary, the prevailing number expressed by EF may rise or decline, but the primary determinant (see eq. (2)) of the actual value is always ESV. When considering the ESV-EDV-domain, it is straightforward to formulate conditions that apply to the newly defined syndrome with pEF referring to a subgroup of HF that must be distinguished from the classical type of systolic HF. The rather flat behavior of EF for the control group underscores the relative importance of ESVI under these circumstances. With the current availability of calibrated values for ventricular volume, there is no need to rely on the EF index, similarly as was done in the past when employing dilution techniques where the index EF emerged as the sole numerical indicator of pump performance. The analysis of a wide spectrum of patient data illustrates that, in particular, ESV exhibits close affinity with EF (Kerkhof 1984, 2000; Kerkhof and Kresh 1990; Beringer and Kerkhof 1998). However, as illustrated in our patient groups, the connection between ESV and EF is not unique but often depends on underlying cardiac pathology.

(B) In another striking example, retrospective analysis of two groups of male patients (Fig. 7) recovering from MI (White et al. 1987), it was shown that the EF versus ESV slope was less steep in patients (*n* = 120) that died from a cardiac cause compared to the other group (*n* = 485). To allow comparison with our presentation (preferring the dependent variable on the ordinate and the independent on the abscissa), we have reproduced a rotated image (Fig. 7) of their original graph. Note that the slope of the line for the patients who died is more shallow. Therefore, values for ESV are different for the two regression lines at any given EF, for example, at EF = 35% (arrow in Fig. 7), except at the point where the lines intersect. The data sets cannot be compared in their entirety because in the MI-study the values for EF were often below 50%. Yet, the graphical findings permit reinterpretation against the background of the ASC framework outlined in the current survey. Additionally, their study documents the superiority of ESV, because multivariate analysis with log rank testing and the Cox proportional hazards model showed that ESV (χ^2^ = 82.9) had greater predictive value for survival than EDV (χ^2^ = 59.0) or EF (χ^2^ = 46.6), whereas stepwise analysis showed that once the relationship between survival and ESV had been fitted, there was no additional significant predictive information in either EDV or EF (White et al. 1987).

(C) We reanalyzed in a similar fashion a published study concerning echocardiographic predictors of outcome in infants with cardiomyopathy (Suda et al. 1997). In four infants idiopathic hypertrophic cardiomyopathy was diagnosed, without associated disease. In another 15 infants, a variety of associated disease was diagnosed, including Pompe's disease, sphingolipidosis, maternal diabetes, and Noonan syndrome. Subdividing these patients into groups surviving or not a 12 months period we found two different regression lines for the ASC:


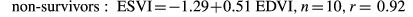



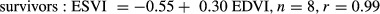


For one infant left ventricular volume (LVV) echo data were not available. Again, the different regression coefficients we derived from their study translate by means of equation 2 into diverging graphs of EF versus ESVI, similar to the ones exemplified in Figure 7. Based on mathematical considerations, a steep slope in the ASC graph generally appears as a shallow regression curve in the EF–ESV representation. This pattern can be appreciated by considering the theoretical case of *r* = 1 in equation 1, reducing further expressions to γ = β and δ = α. Results of these three studies (A, B, and C) are summarized in Table 1.

**Table 1:** Survey of statistical findings, where *n* is number of patients, *P* is significance, n/a means: not available

Label in text	A	B	C
Refers to	This study	White et al. (1987)	Suda et al. (1997)
*n*	34 + 29	485 + 120	10 + 8
Age group	Adults	Adults	Infants
Comparison	pEF versus rEF	(non) survivors	(non) survivors
Figure 4	ESVI–EDVI	n/a	ESVI–EDVI
*P* (slope difference)	<0.0004	n/a	<0.012
Figures 5, 7	EF–ESVI	ESVI–EF	EF–ESVI
*P* (slope difference)	<0.0001	<0.001	<0.034

Critical observations are in order regarding the regression lines encountered in either EF–ESV or ESV–EDV plots. Not only the slope of the regression lines may differ, but also the measure of coherence as reflected by the correlation coefficients *r′* and *r*, respectively. Regarding the latter aspect, we published data which clearly document the inverse linear relationship between EF and ESVI, but emphasized that *r′* is significantly (*P* < 0.02) lower in patients chronically using beta-blockers compared to control patients with coronary artery disease (Kerkhof et al. 1981). Interestingly, also the slope of the ASC for this group was significantly (*P* < 0.001) lower compared to the control group, suggesting that volume regulation of the LV affects the behavior of EF. The fact that EF is not always uniquely related to ESV implies that both variables do not provide equivalent information. From statistical considerations it is clear that ESV(I) or any directly related parameters (such as Emax) form a better discriminator when the regression line of EF versus ESV(I) is relatively flat (Fig. 5). This striking observation is confirmed by the independently formulated results presented in Figure 7 which illustrates that being on a flat or steep regression line appears to be, in fact, a matter of life and death. With the recognition of the newly defined subgroup of HFpEF it becomes evident that the usefulness of EF is rather relative.

Considering the increasing number of reports raising serious concerns about the universal validity of EF, it may prove to be that ESV is the preferable index to evaluate LV function. The intriguing variability of EF in relation to ESV is still in the initial phase of investigation and precludes meaningful reinterpretation of EF until further clarified. It now seems justified to conclude that EF per se has been welcomed by the majority of clinicians, but the occassional or approximate importance of EF as indicator of ventricular pumping capability stems from purely analytical considerations referring to the ESV–EDV domain. The combined information provided by measurement of both ESV and EDV is clearly superior and implicitly includes the derivable value of EF.

### EF as approximate index of ventriculo-arterial coupling

The dimensionless ratio *k* of Emax (the ratio of ESP and ESV, after correction for a dead volume term V_o_) and arterial elastance (defined as end-systolic pressure ESP divided by SV) has been advanced as an index of hemodynamic coupling between LV and arterial system (Kerkhof and Kresh 1990; Marmor et al. 1994; Mathieu et al. 2010). Thus the coupling index can be formulated as *k* = SV/(ESV − *V*_o_). On the basis of equation 1 preload EDV can be replaced by (ESV − α)/β, yielding


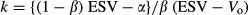
(3)

and indicating that k is a function of α, β, ESV, and V_o_. Also,


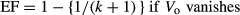
(4)

Thus, EF solely depends on the coupling index *k* if V_o_ = 0, while both EF and *k* are dimensionless as well as ratios of calibrated measurements. Unfortunately, ratios are liable to conceptual shortcomings as mentioned before. For *k* the situation is even worse because *k* is composed of a cocktail of three ratios. Consequently, the use of *k* may imply similar limitations as demonstrated here for EF.

## Discussion

In the past, EF has played a dominant and universally accepted role in the evaluation of ventricular performance, leading to the paradigm that considered EF to be of prime importance. Accepting the novel proposition that EF may not always act as a sensitive indicator opens alternative avenues to analyze LV and RV volume regulation function. This is the first report to document the unique trajectory of ESV versus EDV and the ensuing relationship of EF versus ESV in patients with the newly defined syndrome of HFpEF. It is noteworthy that all patients in our two groups had an EDP in excess of 16 mmHg, while they differed in terms of cut-off points for EDV and EF. Any difference regarding clinical picture (i.e., disease manifestations, spectrum of underlying pathology, possible comorbidities, use of medication, etc.) between the two patient groups would theoretically contribute to the distinct patterns found for the two ESV–EDV lines in Figure 4. However, no preselections of patients were made on the basis of history but assignment to either group was solely based on their LV volumetric characteristics combined with a strict criterion for EDP, in accordance with accepted guidelines (Paulus et al. 2007). These set boundary conditions form the basis for our findings and are sufficient to explain the significantly different slopes for ESV versus EDV as well as those for EF versus ESV. Additional studies are required to analyze the possible modulating effects due to medication, comorbidity, and patient history differences. For example, our earlier investigations on the EF–ESV relationship show that the correlation *r′* is significantly reduced in patients under chronic beta-blockade, leading to a further dissociation between EF and ESV (Kerkhof et al. 1981). Importantly, the linearity of ESV versus EDV still applies under these specific conditions. As we primarily aim to study volume regulation in pEF patients and their matching controls, we clearly preferred to obviate this potential confounding factor.

The ESV–EDV representation is fundamentally a more precise framework for describing LV volume regulation, and as such it does not negate the importance of Starling's law. Notably, in the transformation where SV (in Fig. 1) is replaced by ESV along the ordinate (yielding Fig. 4), one can easily recognize that the new graph implicitly incorporates the classical Starling curve representation. SV can now be derived as the distance between the identity line (not shown in Fig. 4) and the actual regression line (Kerkhof and Kresh 1990; Beringer and Kerkhof 1998). The relatively flat regression line (as found for pEF) implies that this “distance” (and thus SV) increases to a larger extent with elevated EDV compared to a regression line which is steeper. As a consequence, the Starling mechanism is virtually absent if the slope of the ESV–EDV regression line would be exactly equal to 1, that is, a slope parallel to the identity line.

Detailed analysis in terms of P–V relations are required to provide greater insights into the systolic, diastolic, and overall pumping characteristics of the heart. The necessity to measure P in addition to V has been emphasized for describing both LV and RV function. For example, the contractility index Emax depends on ESP and ESV. However, the end-systolic pressure–volume relationship is generally nonlinear, thus complicating detailed interpretation. Furthermore, multiple measurements under different loading conditions are required but not easily performed in critically ill patients. Left ventricular pressure (LVP) measurements are obtained via invasive procedures in contrast to modern LVV determinations. LVP is rather constant in comparison with volumetric alterations, even when LVP is abnormal such as in the case of arterial hypertension or hypotension. Thus, as a first approximation, noninvasive volume measurements such as ESV and EDV may be preferable. This report emphasizes that information on ESV and EDV can be of vital importance, for example, in discerning patients with HFpEF, without the necessity to invoke invasive methods.

Another crucial finding described in the present analysis is the almost linear relationship between EF and ESV(I), also documented by others, yet without any connection to the ASC framework (White et al. 1987). From a clinical perspective it is a challenge to explain the remarkable findings associated with the second (B) and third (C) study (White et al. 1987; Suda et al. 1997), namely why will a patient die, if located on one curve in the EF–ESV plane, in contrast to a nearby potentially achievable state on the curve representing survivors (Fig. 7)? In the study by White et al. (1987), this difference in outcome was a mere observation. Interestingly, we were able to confirm this somewhat bizarre outcome in the patients described by Suda et al. (1997). Within this context we signal the similarity in the case of the pEF group where patients with the “new” type of HF unfortunately are refractory to the standard therapy which as a rule is beneficial to patients with the “classical” type of HF with rEF (Norman et al. 2011).

Two further remarks are in order: one points to the value of correlation *r′* and the other to the steepness of the slope. First, the commonly encountered *r′* with a value well below 1.0 signifies that EF and ESV(I) are not equivalent. It remains to be seen which one is superior and under which circumstances. The second remark refers to the slope of the regression line. Our control group (with EF <50%) exhibits a relatively shallow slope (Fig. 5) similar to the nonsurvivors in another study (Fig. 7) and it is evident that EF is relatively constant compared to the wide spread of data points for the corresponding ESV(I). Irrespective of the underlying cause, when the relationship between EF and ESV is rather flat, it is obvious that ESV is a better discriminator and therefore the parameter of choice. This applies especially to the higher ESV range where EF due to the nonlinearity reported in equation 2 tends to become asymptotic. Various studies have highlighted the importance of ESV(I) (Kerkhof 1984; White et al. 1987; Beringer and Kerkhof 1998). Further clinical studies are required to evaluate the potential and pivotal role of ESV(I) when assessing ventricular performance, for example with the use of the ASC presented in this survey.

In summary, the quantification of EF explicitly depends on ESV, ESP, V_o_ among other factors (see eq. (4)) which may be reformulated as coupling index (see eq. (3)) (Kerkhof and Kresh 1990; Marmor et al. 1994; Mathieu et al. 2010). Simply said: EF embodies a little bit of everything and that notion may explain why it works every now and then, but its clinical utility inevitably faces certain limitations. It is precisely these limitations that are the impetus for consideration of the advanced insights (referring to volumetric regulation based on ESV vs. EDV) in the routine examination of cardiac patients. Our advocacy of studies emphasizing LV internal dimensions (rather than focus on EF) should be explored and finds further support in a recent editorial on the contributions of radial wall thickness and long-axis shortening in HFpEF patients (Manisty and Francis 2008). In addition, a preliminary study based on Monte Carlo simulation regarding LV volume attainable ranges indicates that ESV rather than EDV is the major determinant of EF both in pEF and in HF patients with rEF (Kerkhof et al. 2013).

## Conclusions

The LV volume regulation approach proposed employs primary determinants (such as ESV and EDV) rather than derived quantities (e.g., the ratio EF or its differential parameter SV) as is often preferred by many in the field. The proposed framework affirms a distinct advantage over the classical Starling curve. In addition, we formulated a mathematical expression to describe not only the nearly linear relationship between EF and ESV, but also its asymptotic region. Depending on the particular patient group studied, we observed differing relationships. This observation implies that EF cannot serve as a simple universal indicator of LV performance. We propose that a volume regulatory state diagram in terms of ESV versus EDV forms the cornerstone for more insightful analysis of ventricular function. Such a representation clarifies why some patients exhibit characteristics typical for pEF while others belong to a different group (such as rEF) with inevitably a different prognosis.
