# Left ventricular volume regulation in heart failure with preserved ejection fraction

**DOI:** 10.1002/phy2.77

**Published:** 2013-09-20

**Authors:** Peter L M Kerkhof, J Yasha Kresh, John K-J Li,, Guy R Heyndrickx

Physiol Rep, 1 (2), 2013, e0007, doi: 10.1002/phy2.7

On page 3, the text following equation (2) is incorrectly shown as:

where yielding *δ* = *α* − EDV_ave_(1 − *r*^2^)*β*/*r*^2^

This text should appear as follows:

where *γ* = *β*/*r*^2^ and *δ* = *α* − EDV_ave_(1 − *r*^2^)*β*/*r*^2^

The publisher apologizes for this oversight.

